# Normative data for highly educated older adults in phonemic and semantic fluency tests

**DOI:** 10.1590/1980-5764-DN-2022-0061

**Published:** 2023-05-29

**Authors:** Julianna Pinto de Azevedo, Katie Moraes de Almondes

**Affiliations:** 1 Hospital Universitário Onofre Lopes, Serviço de Neuropsicologia do Envelhecimento, Natal RN, Brazil.; 2 Universidade Federal do Rio Grande do Norte, Natal RN, Brazil.

**Keywords:** Cognition, Executive Function, Language, Elderly, Neuropsychological Tests, Cognição, Funções Executivas, Linguagem, Idoso, Testes Neuropsicológicos

## Abstract

Verbal fluency tests are common instruments used in neuropsychological evaluation and screening for cognitive decline. Different studies have suggested normative data for these tests, but new studies that focus on different educational backgrounds are important due to the heterogeneity of the Brazilian population and the influence of educational level on verbal fluency tests.

**Objective:**

The present study aimed to provide normative data on verbal fluency tests for highly educated older adults in Brazil, as well as the influence of sex, age, and education on test performance.

**Methods:**

A total of 147 healthy volunteers (106 females and 41 males) with a mean age of 66.87 years (SD=4.52) and a minimum of 12 years of education were selected from the community and asked to perform three tests of phonemic verbal fluency (letters F, A, and S) and two tests of semantic verbal fluency (animals and fruits). Volunteers were categorized by educational level into two categories: “High School” (12 years of formal education) and “Higher Education” (over 12 years of formal education).

**Results:**

Normative data are presented in mean values and percentiles for all tests. The performance in animals, fruits, A, and S were associated with educational background. The performance in S was associated with sex.

**Conclusions:**

This study provides normative data appropriate for highly educated, healthy older adults in commonly used tests that evaluate executive functioning. The results endorse previous study findings on the influence of educational level on verbal fluency tests.

## RESUMO.

Testes de fluência verbal são instrumentos comuns em avaliação neuropsicológica e rastreio de declínio cognitivo. Diferentes pesquisas sugeriram dados normativos para esses testes, mas novos estudos com foco em diferentes níveis de escolaridade são importantes diante da heterogeneidade da população brasileira e da influência da escolaridade em testes de fluência verbal.

**Objetivo:**

O presente trabalho teve como objetivo fornecer dados normativos em testes de fluência verbal para idosos brasileiros com alta escolaridade, bem como sobre a influência de gênero, idade e nível educacional nos resultados dos testes.

**Métodos:**

O total de 147 voluntários saudáveis (106 mulheres e 41 homens) com idade média de 66,87 anos (*standard deviation* — SD=4,52) e mínimo de 12 anos de escolaridade foi selecionado da comunidade, e solicitou-se que respondesse a três testes de fluência verbal fonêmica (F, A e S) e dois testes de fluência verbal semântica (animais e frutas). Os voluntários foram categorizados por nível educacional em duas categorias: “Ensino Médio” (12 anos de educação formal) e “Ensino Superior” (mais de 12 anos de educação formal).

**Resultados:**

Dados normativos são apresentados em médias e percentis para todos os testes. A *performance* em animais, frutas, A e S foi associada ao nível de escolaridade. A *performance* em S foi relacionada ao gênero.

**Conclusões:**

Este estudo fornece dados normativos apropriados para idosos com alta escolaridade em testes comumente utilizados na avaliação de funcionamento executivo. Os resultados obtidos corroboram achados de pesquisas anteriores que apontam para a influência da escolaridade em testes de fluência verbal.

## INTRODUCTION

The population of older adults in Brazil is increasing, as data from the Instituto Brasileiro de Geografia e Estatística^1^ show that 10.49% of the Brazilian population is 65 years or older, with an expectation of becoming over 25% of the total population by 2058^1^. Life expectancy also increases in Brazil, as children born in 2022 are expected to live 77.19 years^1^.

The natural aging process includes functional and anatomical changes that modify the capacity of adaptation to the environment, as well as increase the incidence of illnesses^2^ including dementia prevalence^3^. Cognitive functioning is known to naturally change as age increases, and instruments that aim to screen for the presence of cognitive decline have become increasingly relevant^3^ in the clinical evaluation of the elderly population, as they help clinicians differentiate between natural and pathological changes.

Verbal fluency tests are well-known instruments for evaluating cognitive functioning and neurological damage^4^ and are frequently part of screening instruments for older adults^5^. Many versions of these tests are found in the literature, varying on semantic categories or letters used. Generally, these tests consist of evoking words in an established time of 60 s. The test can be of free verbal fluency, with no specific category to the evoked words, as well as phonemic verbal fluency (words that start with a certain letter) or semantic verbal fluency (words that fit within a certain semantic field)^6^. Most of the current literature is regarding the animal category and the letters F, A, and S^7^.

In Brazil, according to Martins et al.^8^, the verbal fluency test was the second most used test from 2012 to 2016. The popularity of these tests may be explained by their fast application and reliability in evaluating executive functioning and semantic memory^4^. Semantic memory is a basic cognitive process that is defined as the memory related to general and specific knowledge of names, words, and meanings^9^. Executive functions are a cluster of cognitive processes that allow for task-driven behavior, planning, and evaluation of behavior efficiency^9^ and are thought to be responsible for integrating basic cognitive processes^4^, therefore playing an important role in functionality. Verbal fluency tests are considered efficient in evaluating these processes as they demand the development of non-habitual strategies, an efficient organization of verbal retrieval, self-monitoring, self-initiation, and inhibition of inappropriate responses^4^.

Previous studies have found an impact of educational level on the results of verbal fluency tests^2,6,7,10–16^, and different normative data and cutoff points have been suggested in the literature. Yet, new normative studies are important, especially due to the heterogeneity of the Brazilian population. Data from the Brazilian demographic census show that 43.7% of individuals aged 25 years or above had 11 or more years of formal education^17^. As some previous studies aim to provide norms to general elderly populations, their sample usually focuses on providing norms for lower educational levels or does not differentiate between individuals with a high school diploma (the equivalent of 12 years of education) from individuals who had attended university. Esteves et al.^6^ provided norms for verbal fluency tests for elders with up to 8 years of education. Machado et al.^2^ and Carvalho and Caramelli^10^ have also provided norms for verbal fluency tests with an educational cluster of 12 years or more. Brincker et al.^12^ analyzed the performance of highly educated older adults in the semantic verbal fluency tests of fruits and animals, yet their study had a small sample (31 individuals) and did not aim to provide normative data for this population.

Additionally, the previous studies were performed in different Brazilian geopolitical regions. Most of the studies have samples based on the Southeast region^2,7,10,11,13^. Esteves et al.^6^ involved a sample of 521 subjects who were residents of the South, and Brincker et al.^12^ involved a sample of 31 subjects from the Midwest. Only the samples in Passos et al.^15^ and Bertola et al.^16^ included subjects from the Northeast, as both studies shared the ELSA-Brasil sample, which is a large sample with 15,105 subjects including those from the Southeast and the South. In the Bertola et al.^16^ study, only data from 9618 participants of the ELSA-Brasil sample were included, as they applied more strict exclusion criteria aiming to provide quality normative data for a brief neuropsychological battery that includes fluency animals and fluency letter F. A total of 12.9% of their final sample was composed of subjects from the Northeastern capital of Salvador. As Brazil is a country with marked social inequalities and economic disparities, results from some geopolitical regions may not be representative of other areas. Specifically, studies carried out with volunteers from the Southeast and South may not be representative of Northeastern elders, which may lead to misdiagnoses of cognitive impairment in this region.

Therefore, this study aimed to provide normative data for highly educated (12 or more years of formal education) healthy older Brazilian adults in semantic and phonemic verbal tests and evaluate the influence of sex and education on test results.

## METHODS

### Subjects and procedure

This study was conducted with 147 healthy volunteers recruited from the community, aged 60–90 years, with 12 or more years of formal education. Exclusion criteria were as follows:

- Less than 12 years of formal education;
- Previous diagnoses of dementia or mild cognitive impairment;
- >5 scores on the Pfeffer Functional Activities Questionnaire^18^;
- Psychiatric conditions without pharmacological management; and
- History of stroke, heart attack, or cardiac arrest in the past year.

Before being invited to data collection, all volunteers underwent a clinical evaluation with a geriatrician to ensure inclusion criteria were met. All volunteers were considered cognitively healthy based on clinical evaluation and results obtained from the Pfeffer Functional Activities Questionnaire^19^ and the Telephone Interview of Cognitive Status (TICS-M)^20^.

Data were collected between March and July 2021 at the Institute of Tropical Medicine, Federal University of Rio Grande do Norte. Participants were assessed through an interview that lasted for approximately 2 h as part of a broader study.

### Instruments

#### Semantic fluency test – animals

Subjects were asked to name as many animals as possible in 1 min. Subjects were informed to name as many animals as they could remember in the time given, regardless of the type of animal, environment, or other characteristics, except for sex variance, and to avoid repeating already named animals. The final score included any valid animal named once.

#### Semantic fluency test – fruits

Subjects were asked to name as many fruits as possible in 1 min. Subjects were informed to name as many fruits as they could remember in the time given, regardless of the type, and to avoid repeating an already named fruit. The final score included any real fruit named once.

#### Phonemic verbal fluency test

The application followed Machado et al.^2^ procedure. Subjects were asked to name as many words as possible with the letters “F,” “A,” and “S” in 1 min for each letter. All subjects underwent the test in the same letter order. Subjects were instructed not to include proper nouns or the same word with a different suffix. The final score for each letter only included correct words named once.

### Statistical methods

The Shapiro-Wilk test was used to verify the normality of the sample. The Mann-Whitney U test was used for group comparisons to analyze age, sex, and educational level. Spearman's rank correlation coefficient between test results and age was performed. Chi-square test was used to test for dependency of the nominal sociodemographic variables. The level of significance considered was p<0.05. Means and standard deviations were calculated for sociodemographic data and test results.

All analyses were performed using the SPSS, version 25.0 software.

### Ethics

This study was approved by the Research Ethics Committee of the University Hospital Onofre Lopes, Natal, Brazil, a member of the National Research Ethics Committee, under the process number 44011221.8.0000.5537. Written informed consent was obtained from all participants before conducting the study. The information collected was kept confidential.

## RESULTS

A total of 147 participants were enrolled in the study. The sample was categorized by two educational levels: “High School degree” (equivalent to 12 years of formal education) and “Higher Education” (above 12 years of formal education). Table 1 shows the mean age and sex, income, and occupational status distribution for the general sample and educational categories. The chi-square test has shown sex and education to be independent variables (X²(1)=1.955; p=0.162). Educational groups had a significant difference in income (X²(6)=43.497; p=0.000) as the higher education group had a higher income. The age range in the sample was 60–78 years. The Mann-Whitney U test has shown a significant difference in results in TICS-M between the two educational levels (U=1010.00; p=0.000). The Mann-Whitney U test has not shown a significant difference in age between the two educational levels (U=1938.00; p=0.920). All total scores had a non-normal distribution.

**Table 1:** Demographic characteristics and clinical data of the 147 subjects sample.

Demographic characteristics and clinical data		General sample (n=147)	High school degree (n=35)	Higher education (n=112)	p-value
Age	Mean±SD	66.86±4.52	66.80±4.49	66.88±4.55	0.920
Sex	Male (%)	41 (27.9%)	13 (37.1%)	28 (25%)	0.162
	Female (%)	106 (72.9%)	22 (62.9%)	84 (75%)	
Income*	R$1,000 to R$1,600	3 (2%)	2 (5.7%)	1 (0.9%)	0.000
	R$1,600 to R$3,000	15 (10.2%)	10 (28.6%)	5 (4.5%)	
	R$3,000 to R$5,000	21 (14.3%)	12 (34.3%)	9 (8%)	
	R$5,000 to R$10,000	35 (23.8%)	6 (17.1%)	29 (25.9%)	
	R$10,000 to R$15,000	32 (21.8%)	1 (2.9%)	31 (27.7%)	
	R$15,000 to R$23,000	27 (18.4%)	4 (11.4%)	23 (20.5%)	
	Over R$23,000	11 (7.5%)	0 (0%)	11 (9.8%)	
	Did not inform	3 (2%)	0 (0%)	3 (2.7%)	
Occupational status	Retired	95 (65.1%)	21 (60%)	74 (66.1%)	
	Unemployed	8 (5.4%)	5 (14.3%)	3 (2.7%)	
	Employed	38 (26%)	8 (22.9%)	30 (26.8%)	
	Did not inform	5 (3.4%)	0 (0%)	5 (4.5%)	
TICS-M	Mean±SD	27.45±4.37	24.90±3.82	28.20±4.25	0.000

Abbreviations: SD: standard deviation; TICS-M: Telephone Interview of Cognitive Status.

* Notes: Income measured in Brazilian Reais (for reference, a minimum wage is R$1,212). Significant results (p<0.05) in group differences are shown in bold.

The total score of both animals (U=1470.500; p=0.026) and fruits (U=1244.50; p=0.007) as well as the total words for letters “A” (U=1483.00; p=0.034) and “S” (U=1497.00; p=0.040) showed significant difference between the two educational level groups. The total words for the letter “F” (U=1856.500; p=0.693) and the sum of all letters “FAS” (U=1555.500; p=0.076) had no significant difference between educational level groups.

Analyses revealed a significant difference in sex groups only for the total number of words for the letter “S” (U=1707.500; p=0.049), with male subjects generating more words than females. When correcting for educational level, the significant difference in sex remains in the high school group (U=54.500; p=0.002), but not in the higher education group (U=1053.000; p=0.457).

There was no correlation between age and any of the total scores.

Test results are described in Table 2 for the general sample, Table 3 for the high school category, and Table 4 for the higher education category. Test results are described in means, standard deviations, and percentiles. Due to the non-normal distribution of scores, the use of percentiles is recommended when evaluating cognitive decline. Table 2 results are recommended as norms for letter F and fluency FAS results, as these tests showed no significant difference between educational levels.

**Table 2:** Normative data for 60–78 years old with 12 or more years of formal education.

General sample (n=147)								
Test	Mean±SD	P90	P75	P50	P25	P10	P5	P2
Fluency animals	18.27±4.93	25	21	18	14	13	11	9
Fluency fruits	16.53±3.74	22	19	16	14	11	10	8
Letter F	12.25±4.73	19	16	12	9	6	5	4
Letter A	12.56±4.56	19	16	12	10	6	6	4
Letter S	12.79±3.96	19	16	12	10	8	7	4
Fluency FAS	37.61±11.72	54	46	37	29	22	19	17

Abbreviation: SD: standard deviation.

**Table 3:** Normative data for 60–78 years and 12 years of formal education.

High school (n=35)								
Test	Mean±SD	P90	P75	P50	P25	P10	P5	P2
Fluency animals	16.52±4.35	22	20	17	13	10	8	8
Fluency fruits	15.00±3.70	20	17	15	13	10	8	6
Letter F*	11.76±4.70	19	14	12	8	5	4	4
Letter A	11.02±4.47	18	14	11	9	5	4	4
Letter S	11.55±3.61	16	14	12	9	7	6	5
Fluency FAS*	34.35±10.86	51	46	3	27	19	17	16

Abbreviation: SD: standard deviation.

* Note: No significant difference between educational levels.

**Table 4:** Normative data for 60–78 years old with more than 12 years of formal education.

Higher education (n=111)								
Test	Mean±SD	P90	P75	P50	P25	P10	P5	P2
Fluency animals	18.73±5.10	25	21	19	15	13	12	10
Fluency fruits	17.02±3.64	22	19	17	14	12	11	9
Letter F*	12.40±4.75	19	16	12	9	6	5	4
Letter A	13.06±4.50	20	16	12	10	7	6	6
Letter S	13.19±4.00	19	16	13	11	9	7	4
Fluency FAS*	38.66±11.84	55	45	38	29	23	21	18

Abbreviation: SD: standard deviation.

* Note: No significant difference between educational levels.

## DISCUSSION

The objective of the present study was to provide normative data for the phonemic and semantic verbal fluency tests for a population of highly educated, healthy older adults. Our study presents data from a sample of community volunteers in this specific population from the northeastern region of Brazil. We also aimed to investigate differences in test results related to educational background and sex.

We found no association between test performance and age. Other studies have controversial findings when analyzing the correlation between age and performance in verbal fluency tests. Zimmermann et al.^14^ found age to be related to both a phonemic task using the letter P and semantic task of 2 min for naming articles of clothing in a study with 300 adults aged from 19 to 75 years. Esteves et al.^6^ have found an association between age and performance in FAS, but not in the animals category in a sample of elders with up to 8 years of education. Carvalho and Caramelli^10^ had similar findings in a sample aged between 45 and 64 years and 4 to 12+ years of formal education, as they found age to be correlated with FAS and letter F, but not with letter A or S or animals. In contrast, Machado et al.^2^ did not find an association between age and FAS performance in a study with 345 elderly individuals with 1–24 years of formal education. Brincker et al.^12^ also did not find an association between age and performance in semantic verbal fluency tests in their study with 31 highly educated (8–25 years of formal education) elders. The lack of association between age and test performance may be explained by the small age variation, as well as the educational characteristics of the sample, as educational level may have compensated for age effects. Previous studies have shown education to have a greater impact on both semantic and phonemic verbal fluency tests^2,14,16^.

In our study, we found an association between sex and phonemic verbal fluency for the letter S, with men generating more words than women. When analyzed by educational groups, the association remains only in the high school group. This suggests that such differences may be explained by differences in educational level and income. Similar findings have been described for animal fluency in previous studies^12^. Generally, sex differences in verbal fluency test results are explained by cultural background and differences in social roles^12^. However, other studies have found no correlation^2,6,7,16^. These findings may also be explained by imbalance between sex in the sample.

Education has a well-known influence on verbal fluency tests. Machado et al.^2^ have found education to be the more important variable in predicting FAS results. Zimmermann et al.^14^ found education to have an independent effect on participant's age in both semantic and phonemic tasks. Brucki and Rocha^7^ found the educational level to affect the total number of animals in semantic verbal fluency test, as well as the number of categories of animals and changes in categories and clusters. Bertola et al.^16^ found education to be more correlated than age with cognitive scores on a brief neuropsychological battery that includes fluency animals and fluency letter F in their sample of ages 35–74 years. Even in highly educated samples, such as the one used by Brincker et al.^12^, significant differences are found in performance on both animals and fruits semantic verbal fluency tests, with individuals with 17–25 years of schooling performing better than those with 8–16 years. These findings show the importance of normative data for different educational backgrounds, as verbal fluency tests are highly affected by this variable. We have chosen to divide our sample into two educational clusters commonly used in clinical practice, as a high educational background is often registered as “completed high school” or “completed or incomplete high education”.

This study has limitations, such as an imbalance between educational level categories and sex. We chose to register educational level as a nominal variable instead of measuring years of formal education, therefore limiting the statistical analysis. A small variation in ages in our sample, as the minimum age was 60 years and the maximum age was 78 years, may explain the lack of association between age and test performance. Another limitation is the possibility of selection bias due to the community-sourced volunteer sample. To reduce such bias, we applied exclusion criteria and screened volunteers for previous cognitive decline.

This study provides normative data for healthy older adults on phonemic verbal fluency test (FAS) and two semantic verbal fluency tests (animals and fruits). These normative data are fitted for highly educated, healthy Brazilians aged 60–78 years. We provide both means and percentile scores for two categories of educational background and the total sample. This study aims to help clinicians in the assessment of patients and may help increase precision in the evaluation of cognitive decline in this specific population.
